# Ultrahigh strength ductile microalloyed steel with a very low yield ratio developed by quenching and partitioning heat treatment

**DOI:** 10.1038/s41598-022-11722-7

**Published:** 2022-05-13

**Authors:** S. Fida Hassan, H. AlWadei

**Affiliations:** 1 Department of Mechanical Engineering, King Fahd University of Petroleum and Minerals, Dhahran, 31261 Saudi Arabia; 2 Interdisciplinary Research Center for Advanced Materials, King Fahd University of Petroleum and Minerals, Dhahran, 31261 Saudi Arabia

**Keywords:** Engineering, Materials science

## Abstract

In this study, a microalloyed low carbon steel was subjected to quenching and partitioning (Q&P) heat treatment processes. The primary ferrite-pearlite microstructure of the steel was transformed into a bainitic microstructure containing interlath and sporadic blocks of retained austenite. The applied heat treatment process partitioned the carbon into the retained austenite to a weight percentage of 0.136. The microalloyed low-carbon steel acquired a continuous yield with high yield strength, a gigapascal level of ultimate tensile strength (i.e., ~ 1.1 GPa), and a very low yield ratio (i.e., 0.55) while retaining reasonable ductility and toughness when compared to the preheat-treated values.

## Introduction

Microalloyed steels are in high demand in industries, including oil and gas transportation sectors as well as automobile industries. Alloying elements affects the phase transformation behavior during processing and ensues better mechanical properties^1–3^. Demand by the oil and gas transportation pipeline industry for steel with better mechanical properties, mainly strength and toughness, for more effective and efficient oil and gas transportation has led to the development of a series of pipeline steels. These mechanical properties of steels are derived from the manipulation of microstructure through composition optimization^4,5^, thermomechanical processing^6,7^ and heat treatment^8–11^. Thermomechanical and/or heat treatment processing transforms the ferrite-pearlite banded microstructure into various phases and/or morphologies, including polygonal ferrite, quasi-polygonal ferrite, granular-bainitic ferrite, bainitic ferrite and acicular ferrite^2,12^. Among these polygonal ferrite (recrystallized smooth boundary large equiaxed grain) is the softest, while acicular ferrite (fine grain with islands of quasi-polygonal, granular bainitic ferrite and bainitic ferrite) is the strongest with reasonable ductility. Although in these cases the processed microstructure typically contains more than one of the phases. The heat treatment process involves heating for partial or full austenization followed by a continuous or isothermal quenching to a lower temperature for projected phase transformation. Quenching and partitioning (Q&P) is a relatively new concept of heat treatment^13^, where the fully or partially austenized steel is quenched to martensite formation temperature and continues to anneal to facilitate carbon partitioning from martensite to retained austenite without carbide formation. Carbon enriched austenite stabilizes at room temperature and consequentially induce simultaneous improvement in the strength and ductility of the steel. This quenching and partitioning procedure was initially proposed and then widely used on medium carbon steel and was latter also applied to low carbon transformation induced plasticity steel^14^ and pipeline steel ^11^. In the present study, we used quenching and partitioning heat treatment on microalloyed low carbon pipeline grade steel API X65 in order to study the effect on microstructure and mechanical properties.

## Results and discussion

**Figure 1:**
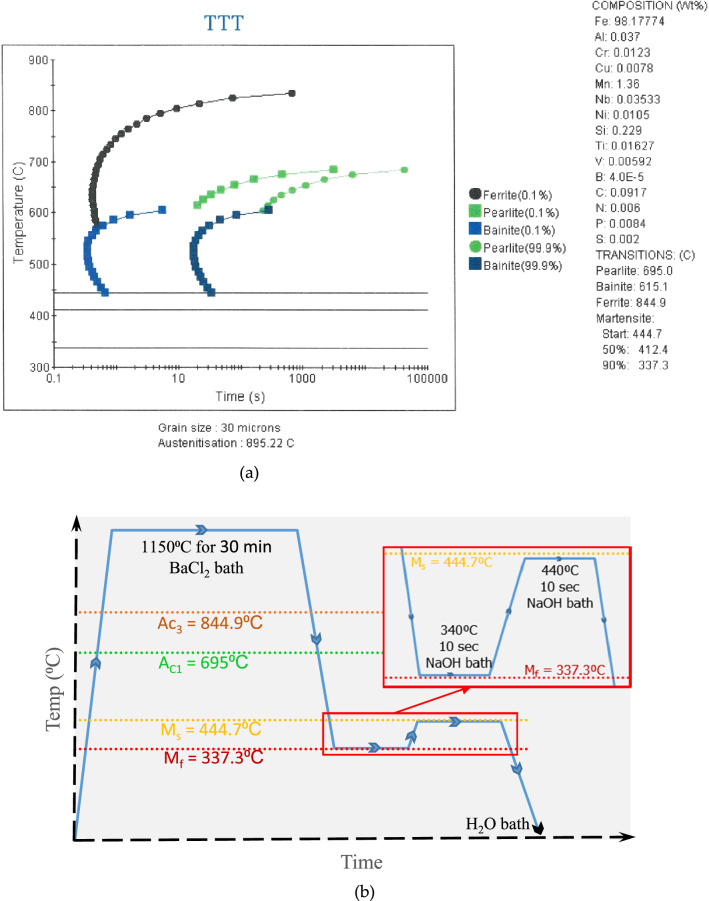
(**a**) Time–Temperature-Transformation diagram for the microalloyed steel, and (**b**) the quenching and partitioning heat-treatment cycle used in this study.

The carbon equivalent for the low carbon microalloyed steel (see Table 1) was calculated using the ItoBessyo formula^15^ to a value of 0.168. It should be noted that the Ito-Bessyo formula $\left\{ {{\text{i}}.{\text{e}}.,{\text{ CE}}_{{{\text{P}}\left( {{\text{CM}}} \right)}}  = {\text{C}} + \frac{{{\text{Si}}}}{{30}} + \frac{{{\text{Mn}}}}{{20}} + \frac{{{\text{Cu}}}}{{20}} + \frac{{{\text{Ni}}}}{{60}} + \frac{{{\text{Cr}}}}{{20}} + \frac{{{\text{Mo}}}}{{15}} + \frac{{\text{V}}}{{10}} + 5{\text{B}}} \right\}$ is applicable to calculate the carbon equivalent for steel with a carbon content less than 0.12 weight percentage and it is also called the ‘parameter for cracking measurement’. The martensite transformation start temperature, MS (°C), was estimated using the empirical formulae proposed by Andrews^16^ $\left\{ {{\text{i}}.{\text{e}}.,{\text{M}}_{{{\text{S }}}} \left( {^\circ {\text{C}}} \right) = 539 - 423{\text{C}} - 30.4{\text{Mn}} - 12.1{\text{Cr}} - 17.7{\text{Ni}} - 7.5{\text{Mo}} + 10.0{\text{Co}} - 7.5{\text{Si}}} \right\}$, and Seo et al.^17^ $\left\{ {{\text{i}}.{\text{e}}.,{\text{M}}_{{{\text{S }}}} \left( {^\circ {\text{C}}} \right) = 764.2 - 302.6{\text{C}} - 30.6{\text{Mn}} - 14.5{\text{Si}} - 8.9{\text{Cr}} - 16.6{\text{Ni}} + 2.4{\text{Mo}} - 1.3{\text{Cu}} + 8.58{\text{Co}} + 7.4{\text{W}}} \right\}$, to be 457 °C and 464 °C, respectively. These theoretical values for the martensitic transformation start temperature closely matched the value determined using JMatPro® software. The critical temperatures (see Fig. 1a) for the microalloyed API X65 steel were determined to be 844.9 °C (the upper critical transformation temperature), 695 °C (the lower critical transformation temperature), 444.7 °C (the martensite transformation start temperature), and 337.3 °C (the martensite transformation end temperature).

**Table 1:** Chemical composition of the microalloyed low carbon steel.

C	Si	Mn	P	S	Al	Cr	Ni	Cu	V	Nb	Ti	N	Ca
0.091	0.229	1.36	0.008	0.002	0.037	0.0123	0.011	0.008	0.006	0.035	0.016	0.006	0.002

**Figure 2:**
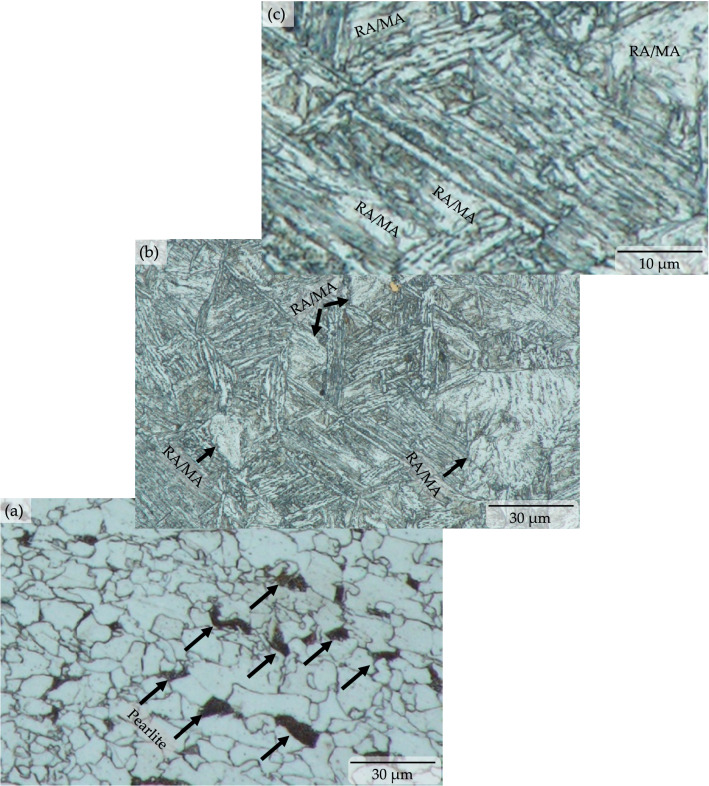
Optical micrographs showing pre-heat treated ferrite-pearlite microstructure of microalloyed low carbon steel [‘black arrow’ marks pointing to the sporadic pearlite colony in the ferrite matrix] (**a**) transformed into bainitic ferrite sheaves with retained austenite and/or martensite/austenite constituent [‘RA’ and ‘MA’ represents retained austenite and block of martensite/austenite constituent and are pointed by ‘black arrow in lath bainitic ferrite matrix] due to quench and partitioning heat treatment (**b**) and (**c**), respectively.

**Figure 3:**
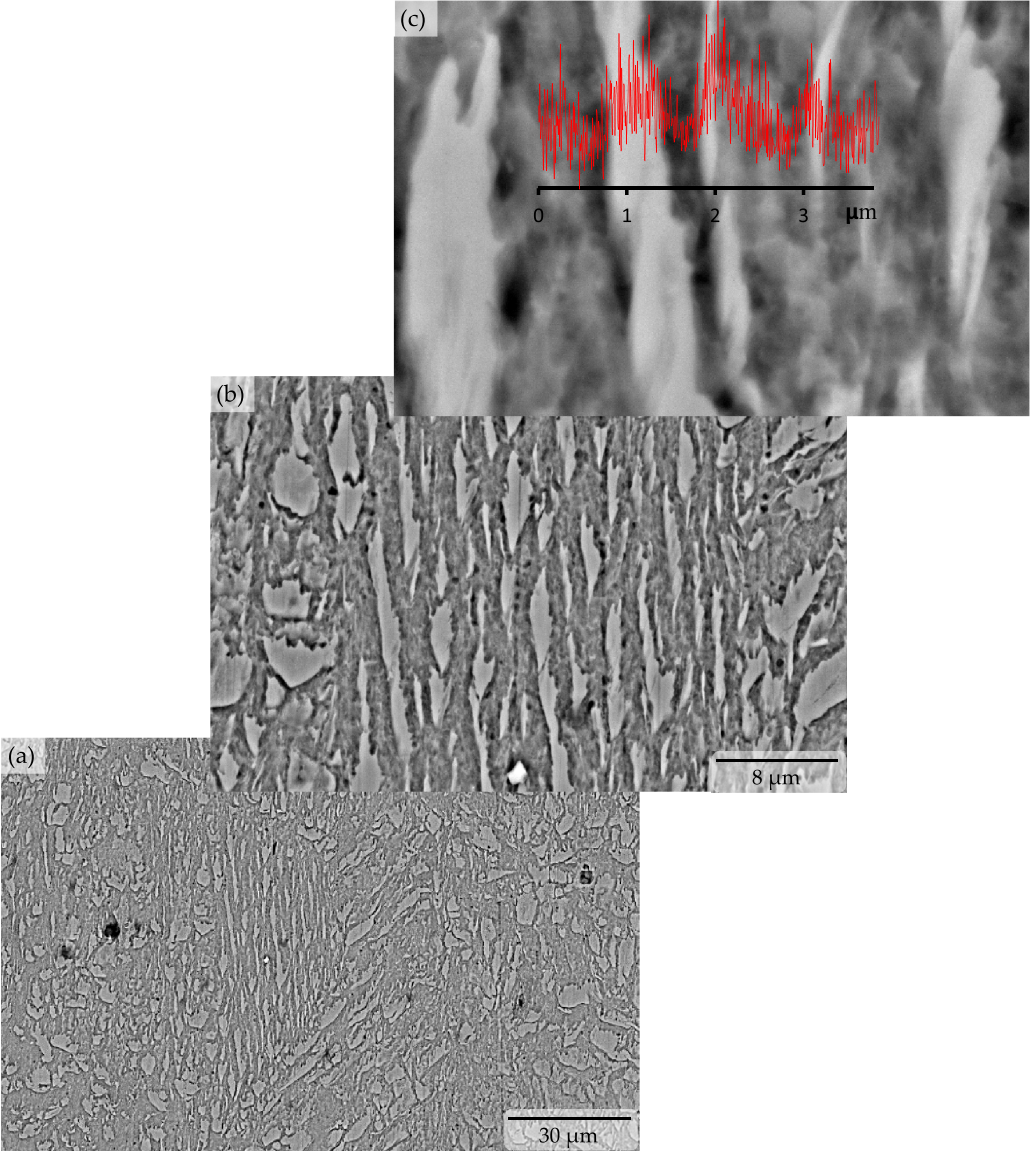
Backscattered electron image showing bainitic ferrite sheaves with retained austenite and/or martensite/austenite constituent due to quench and partitioning heat treatment in different magnification, with carbon concentration profile along the micro-constituents.

The applied quenching and partitioning process effectively transformed the microstructure of primary ferrite with sporadic pearlite (see Fig. 2a) of the microalloyed low carbon steel into lath-bainitic ferrite (BF) sheaves with the presence of significant amounts of retained austenite (RA) and/or martensite/austenite (M/A) constituent blocks (see Fig. 2b, c and 3). The parallel arrangement of the acicular shaped ferrite sheaf in low-carbon steel and/or in low-carbon alloy steel is considered upper bainite by Ohtani^18^, and Bramfitt and Speer ^19^. The salient features of the bainitic ferrite are (i) nucleation at the austenite boundary (see Fig. 2b), (ii) alignment and elongation in parallel form (see Fig. 2b, c), and (iii) their coexistence with the austenite and/or martensite/austenite constituents (see Fig. 2b, c and Figure 3). All of these were observed in the microstructure.

**Figure 4:**
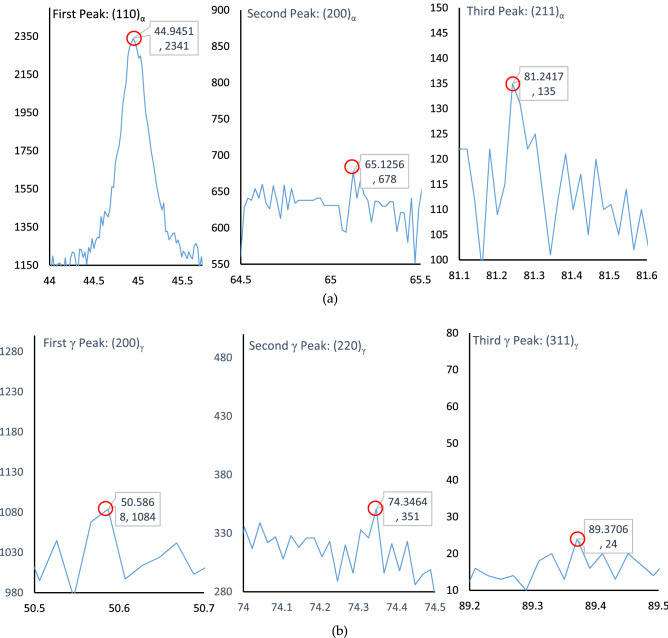
X-ray diffraction peaks of quench and partitioning heat treated microalloyed low carbon steel for ferrite (**a**) and austenite (**b**) used to estimate the amount of retained austenite.

Upon heating, austenization of the microalloyed steel sample microstructure was initiated when heated to 695 °C (i.e., the lower critical or eutectoid transformation temperature) (see Fig. 1) and completed at temperatures above 844.9 °C (i.e., the upper critical transformation temperature). On the other hand, the heating of the sample at 1150 °C (a temperature much higher than the upper critical temperature) caused dissolution of any potential carbides present in the microstructure, except for titanium carbide, and further it homogenized the alloying elements present. Upon quenching, the nucleation of the bainitic ferrite sheaf was initiated at the austenite grain boundaries followed by inward parallel growth. In the case of steel with very low carbon content, typically the upper bainitic ferrite contains retained austenite between the sheaf instead of carbides^2,3,19,20^. The microstructural characterization of the quenched and partitioned low carbon microalloyed steel also revealed the existence of untransformed blocky retained austenite and/or martensite/austenite constituents (see Fig. 2c and 3b, c). This phenomena was also reported in the literature for similar materials^21,22^. Carbon in the high temperature austenite was anticipated to be partitioned during the austenite-bainite transformation throughout the quenching stage, and further carbon migration toward the retained austenite was likely to be experienced throughout the partitioning stage (see Fig. 3c). It should be noted that previous researches did not report any redistribution or partitioning of substitutional alloying element atoms during the bainitic transformation and growth^23,24^. Analysis of the X-ray diffraction results estimated the presence of 19.76 volume percentage of retained austenite with 0.136 weight percentage of carbon content in the quenched and partitioned microalloyed low-carbon steel microstructure. This analysis was conducted using the direct comparison method^22,25^ of X-ray diffraction peaks, where the (200)α, and (211)α X-ray diffraction peaks were compared to the (200)γ, (220)γ, and (311)γ peaks (see Fig. 4) for the samples. It should be noted that the carbon content in the retained austenite was much higher than the initial average alloy composition. This higher carbon content and the presence of other alloying elements stabilized the retained austenite and/or martensite/austenite constituents to room temperature ^1–3,13,14^.

Mechanical characterization of the quenched and partitioned microalloyed low carbon steel estimated a microhardness value of 275 HV (see Table 2). This could be attributed to the presence of a higher dislocation density in the bainitic ferrite and the number of grain boundaries in the microstructure^2,19,20^. However, it should be noted that this microhardness value for this quench and partitioning heat treated microalloyed low carbon steel remained within the upper limit value suggested for offshore and sour applications^26^.

**Table 2:** Mechanical properties of quenching and partitioning heat-treated microalloyed low carbon steel.

Materials	Mechanical properties						
	0.2% YS (MPa)	UTS (MPa)	Yield ratio	Ductility (%)	Energy absorbed		Hardness (HV)
					Tensile (J/mm^3^)	Charpy (J)	
Q&P	594	1082	0.55	18.42	151	130	275
As-received	428	530	0.81	28.70	139	172	211
API X65^26^	450–600	535–760	≤ 0.93	≥ 16	–	≥ 40–68	≤ 275 (sour) & ≤ 270 (offshore)

**Figure 5:**
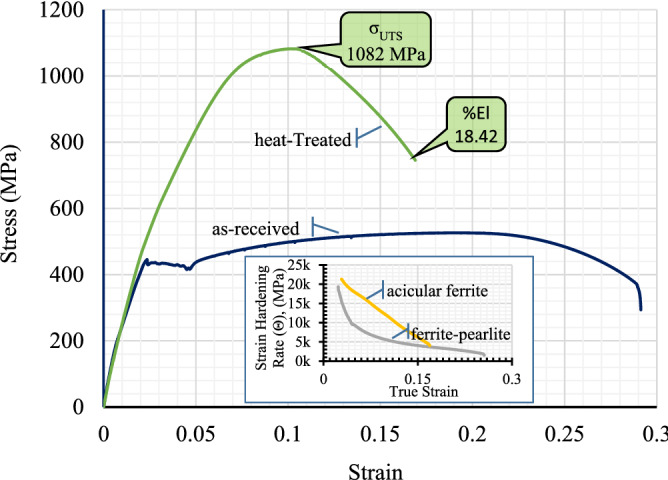
Tensile stress–strain behaviour (inset: strain hardening rate vs true strain) for microalloyed low carbon steel.

Transformation in the microstructure led to the phenomena of continuous yield in the quenched and partitioned microalloyed low carbon steel, which is a major shift from the discontinuous yield of low carbon pipeline steel. In addition, this improvement in the strength of the steel led to an ultimate tensile strength to gigapascal level (i.e., ~1.1 GPa) with a very low yield-to-tensile ratio (i.e., 0.55). However, this quench and partitioning heat treated microalloyed low carbon steel retained the initial ductility to a reasonable level (i.e., 18.42 percentage) sufficient for the oil and gas transportation pipeline application (see Fig. 5 and Table 2).

**Figure 6:**
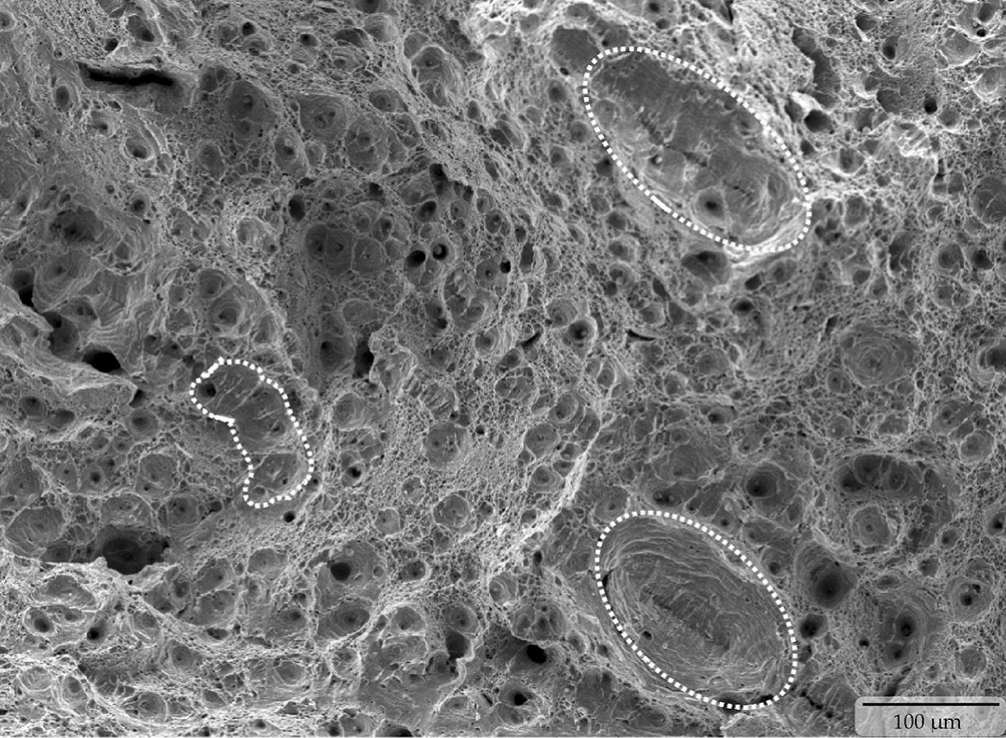
Tensile stress produced ductile fracture with three types of dimples (i.e., fine, coarse and large elliptical crater) in the quenched and partitioning heat-treated microalloyed low carbon steel.

**Figure 7:**
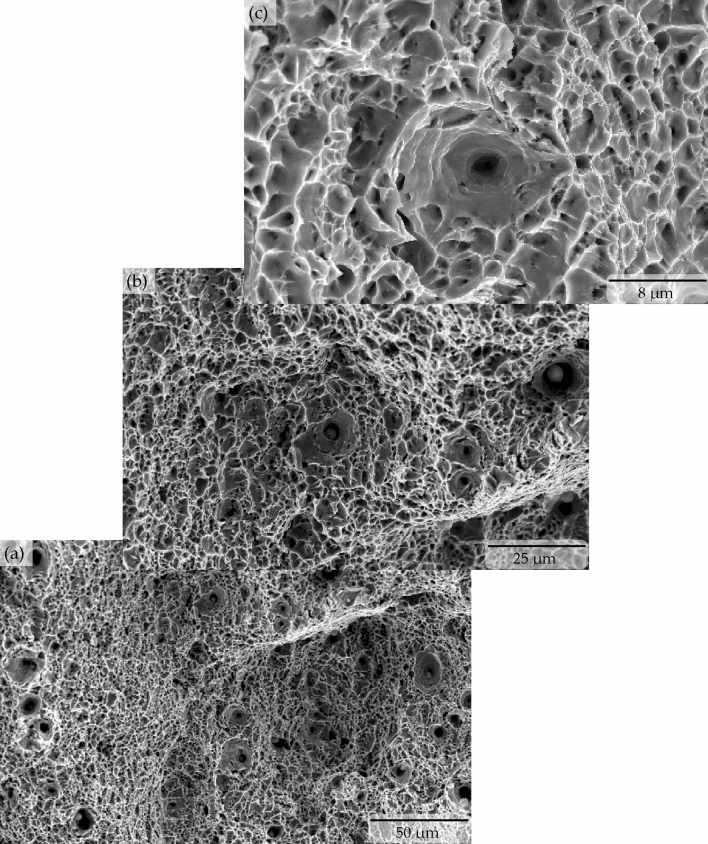
Scanning electron microscopy showing fine dimples dominated ductile fracture surface in pre-heat treated microalloyed low carbon steel.

**Figure 8:**
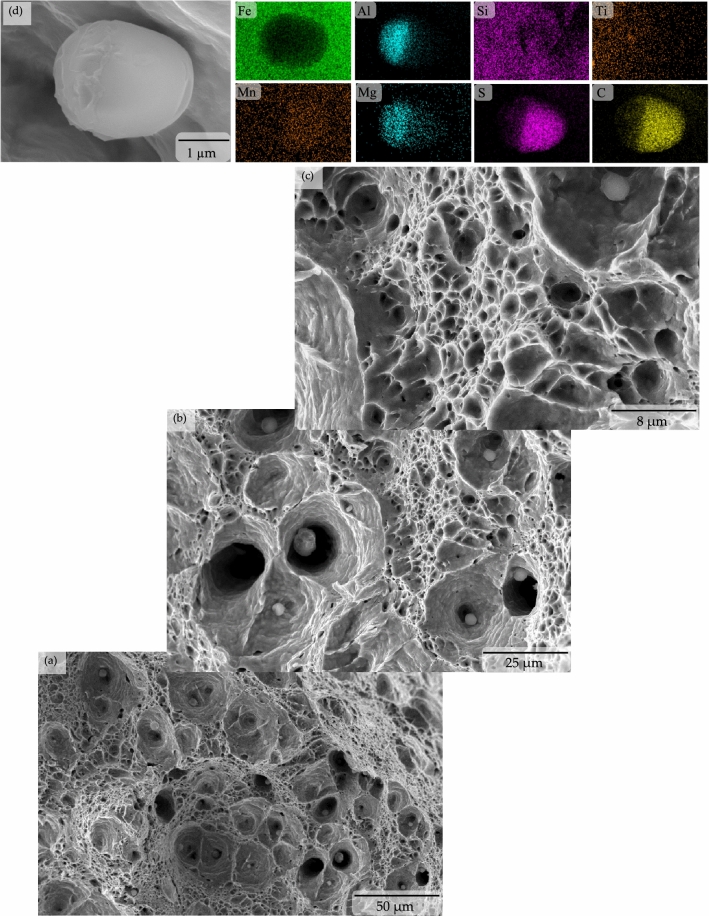
Scanning electron microscopy showing coarse dimples dominated ductile fracture surface in the quenched and partitioning heat treated microalloyed low carbon steel (**a**)–(**c**) caused by the presence of fine metalloids (**d**), respectively.

The superior tensile properties (i.e., both the yield and ultimate tensile strength) were considered to be the results of the microstructural features developed in the steel samples due to the applied quenching and partitioning heat treatment process. The developed microstructural features were also apparently able to stimulate a very large amount of work hardening effect in the microalloyed low carbon steel. Earlier research works in the open literature reported that the inherent presence of a large amount of dislocation in the fine bainite lath is the reason to induce a continuous yield with a significantly high yield strength in a low-carbon steel, which also subsequently triggers an extensive work hardening effect in the material^2,21,27^. The presence of a considerably large amount of retained austenite also provided much higher and incessant strain hardening in the quenched and partitioned microalloyed low carbon steel samples until approximately 11% uniform plastic deformation and resulted into a staggeringly high ~1.1 GPa of ultimate tensile strength. The microvoids in the tensile samples started to nucleate from the presence of (i) extremely fine carbide particles (which led to the formation of fine dimples), and (ii) nonmetallic particles (which led to the formation of large dimples^28^). Microvoids also nucleated in the tensile sample microstructure from decohesion of the bainite sheaves at the interface with block-retained austenite (which led to the formation of massive elliptical and/or elongated craters^28–30^). Presence of all of these three types of dimples and elliptical craters were evident in the quenched and partitioned sample fracture surface as shown in the Fig. 6. The growth of microvoids from the finer size (in the case of the preheat-treated sample, as seen in Fig. 7) to the relatively much larger size dimples of different shapes (in the case of the quenched and partitioned heat-treated sample, as seen in the Figs. 6 and 8) due to their coalescence eventually resulted in the fracture of the sample with an 18.42% elongation.

The monotonic decline in the work hardening rate with increasing true strain (inset of Fig. 4) is an indication of the work hardening induced ductility improvement in the quenched and partitioned heat-treated microalloyed low carbon steel. The significantly low yield-to-tensile strength ratio (i.e., 0.55) is an indication of the spectacular enhancement in the resistance to deformation from the yield point to the plastic instability^27^ of the transformed microstructure of microalloyed low carbon pipeline steel. The room temperature toughness (tensile and impact) of the quenched and partitioned microalloyed low carbon steel remained reasonably high (see Table 2) from the recommended minimum required values. This is despite of the remarkably high strength achieved by the samples due to the quenching and partitioning heat treatment process. In essence, the mechanical properties remained much higher than that required for the oil and gas transportation industries as prescribed by the American Petroleum Institute *s*tandards^26^.

## Conclusion

The quenching and partitioning heat treatment process transformed the microalloyed low carbon steel microstructure to lath bainite with interlath and block retained austenite. The retained austenite stabilized to room temperature due to partitioning of carbon into it. The transformed microstructure assimilated a continuous yield with high yield strength, and strong work hardening which resulted in a gigapascal ultimate tensile strength, ensuing a significantly low yield-to-tensile strength ratio. Despite the remarkable strengthening effect, the quenched and partitioned microalloyed low carbon steel retained reasonably high ductility and toughness.

## Methods

### Materials and heat treatment

A 15.89 mm thick rolled API X65 coil (composition in Table 1) was machined to cylindrical tensile specimens (diameter 6 mm, gauge-length 30 mm) and V-notch Charpy impact test specimens. Samples were heated to 1150 °C for 30 min for full austenization followed by quenching to 340 °C for 10 s, and then partitioning was continued at 440 °C for 10 s prior to quenching to the room temperature. Salt bath was used for the quenching and partitioning heat treatment process. Figure 1 shows the schematic view of the heat treatment cycle used. The critical temperatures (i.e., the upper and lower critical transformation temperatures, and the martensite transformation start and end temperatures) for the microalloyed low carbon steel were determined using JMatPro® software.

### Microstructural characterization

Microstructural characterization of the quenching and partitioning heat treatment processed sample was conducted through optical microscopy (OM) (MODEL: Olympus DXS-510 Digital, Japan), scanning electron microscopy (SEM) (MODEL: Quanta 250 FEG, FEI, Czech Republic) for phase identification and distribution, and X-ray diffraction (MODEL: Bruker AXS D2 PHASER 2nd Gen Diffractometer, USA) for quantification of retained austenite and its carbon content. Samples were metallurgically polished, and etched with 4% nital solution for 5 s. PDXL2 software was used to identify the X-ray diffraction spectrum peaks.

### Mechanical characterization

Mechanical characterization of the quenching and partitioning heat treatment processed samples was conducted for microhardness (MODEL: Beuhler MMT-3, USA) and room temperature elongation-to-fracture tensile properties (MODEL: Instron 5569 Universal Testing Machine, Instron, USA). Microhardness values were measured on metallurgically polished samples in accordance with the ASTM E384-18a standard using a load of 500 gf and a dwell time of 10 seconds. Tensile properties were measured in accordance with ASTM E8/E8 M-16ae1 using a machine cross-head speed of 1 mm.min-1. The room temperature toughness of the heat-treated samples was measured using a notched bar impact test (MODEL: Pendulum Impact Tester PSd 300, WMP Leizig, Germany) in accordance with the ASTM E23-18 standard. Scanning electron microscopy was conducted on the tensile fracture surfaces to understand the fracture mechanisms.
